# The determinants of the choice of treatment of pregnant women in Cameroon

**DOI:** 10.1186/s13561-016-0127-1

**Published:** 2016-10-24

**Authors:** Saturnin Bertrand Nguenda Anya, Atanase Yene

**Affiliations:** Faculty of Economics and Applied Management, University of Douala, Douala, Cameroon

**Keywords:** Choice of treatment, Pregnant women, Maternal health, Nested multinomial logit model

## Abstract

This paper seeks to identify the determinants of the choice of treatment of pregnant women in Cameroon. Theoretically, the methodology is based on a discrete choice model with random utility. Empirically, the econometric specification is a Nested Multinomial Logit Model. The data used comes from the Demographic Health Survey (DHS) organized in 2011 by the National Institute of Statistics. The results reveal that uneducated women or those having only a primary education prefer to meet the traditional midwives than seek modern maternal health services. Moreover, the absence of a paid job for the pregnant woman, the large size of the household, Islamic or animist religion, poverty, high costs of healthcare and transportation are constraints which make the pregnant woman to prefer the services of traditional midwives to modern services of maternal health. The use of modern healthcare services by pregnant women in Cameroon can therefore be improved by at least two means: firstly, by improving on the level of education of women and economically empowering them. Secondly, in a context where the costs of healthcare services are paid directly by the pregnant women themselves or by their families, it is important to put in place health insurance schemes in order to guarantee proper follow-up of pregnant women until delivery as well as taking care of complicated cases.

## Background

The health services received by a mother during pregnancy, at birth and immediately after delivery are not only important for her health and survival but also for those of the child. Health is an element of human capital, as well as education and nutrition. In fact, [1] defines human capital as a set of cognitive, physical, nutritional, and biological aptitudes which reinforce human capacities. Human capital has two main components: education and health. The application of the concept of human capital to health was initially developed by [1], followed by [2] who develops the concept of health-capital. In fact, the notion of health-capital considers health as a perishable good whose stock must be maintained by investments in health. According to the health-capital model, individuals receive a stock of health-capital at birth, which depreciates with time but can be increased through investment; death occurs when the stock of capital falls below a given threshold. For this reason, health services are considered as a necessary input for the production of the optimal stock of health-capital [3]. From this point of view, the non consumption or low consumption of maternal health services constitutes an obstacle to health-capital, hence human capital. Also, with the extension of consumer theory by [4], utility no longer depends only on the quantity of goods but also on the characteristics of these goods. The utility the pregnant woman derives from health services thus depends on the characteristics of the health services. Following this approach, women arbitrate between the alternative sources of treatment according to the characteristics of the maternal health services. In this context, lack of courtesy by the personnel, humiliating and mocking remarks made towards certain women; long queues and sometimes hurried consultations, for example, create a disutility and prevent the recourse to modern health services [5].

There is an abundant literature on the determinants of the usage of maternity services in low or middle income countries. These studies focus on the determinants of the usage of maternal health services [6–9], or on the frequency of utilisation of these services [10–12]. Others focus on the relationship that exists between the utilization of maternal health services and factors like the place of residence [13], the level of education of the mother [8, 14], the wealth index of the household [8, 15], the income of the mother and her ethnic group [14]. However, this empirical literature does not give enough information on the determinants of the choice of treatment of expecting mothers in an environment where a variety of modern health services coexist with those of traditional midwives.

In Cameroon, [16] find that the proportion of the pregnant womens who consult a doctor at least once increases with the level of education. Less educated women less often demand treatment because they lack financial means or are less familiarized with the health system. As the level of pregnant women increases, the more they enter the medical coverage and the more their demand for healthcare is directed towards the medical units. Beninguisse [17] shows that the use of obstetric services by pregnant young women is considered as a function of their perceptions, beliefs and knowledge in the field of obstetrics, their financial resources and the accessibility of the medical units. Also, [18] find that the use of health services depends on social institutions such as habits, solidarity networks, perceptions or symbolic image of pregnancy and childbirth, degree of openness to modernity, and the economic conditions in which the women live. Existing studies on the determinants of the use of maternal health services in Cameroon are limited by the fact that they consider all modern health services as homogenous. However, the perception that a pregnant woman has of a district public hospital is not necessarily the same as that she has of the denominational or private district hospital while in both cases, the hospitals are modern health services. For example, an anxious mother will be able to travel a long distance to treat her child in a private health establishment or traditional midwife in which she has confidence but will not go the few kilometres separating her from the nearest public hospital because she believes that the latter will not be able to serve her properly [19]. This study aims at correcting this limit by seeking the determinants of the choice of healthcare of pregnant women taking into account the specificities of the health services.

The main objective of this study is to identify the determinants of the choice of health services of women in Cameroon. Its main contribution is that it identifies the determinants of the choice of treatment of pregnant women taking into consideration modern maternal health services and traditional ones.

The significance of this study is two fold. Firstly, maternal health is a millennium development objective with the target at 350 deaths per 100.000 live births according to the Growth and Employment Strategy Paper (GESP) [20]. In its GESP, the government in order to achieve this goal provides free treated mosquito nets for pregnant women and a reduction of obstetric costs. The success of this strategy primarily depends on the identification of the determinants of the use of maternal health services. Secondly, in view of reaping the benefits of the demographic transition, particularly the demographic dividend which is sought in the majority of Sub-Saharan African countries and Cameroon in particular, it is important to reinforce mother and child health by reducing maternal mortality. The demographic transition is a change in the demographic trends of the population of a country that results in low fertility and mortality rates. The expected benefit of the demographic transition is the demographic dividend, i.e. the acceleration of economic growth because of an increase in the working population and a fall in the dependent population.

The rest of the paper is organized as follows. Section The state of maternal health services in Cameroon presents the state of the use of maternal health services in Cameroon. Section Methods presents the methodology. Section Results and discussion discusses the results and section Conclusion concludes.

### The state of maternal health services in Cameroon

The question of the access to maternal health services in the Cameroonian context is a major concern for the attainment of the Millennium Development Objectives and the evaluation of public policies of maternal health put in place by the government within the framework of its sectoral health strategy (SSS). In fact, the success of the public policies of maternal health requires the identification of the factors which explain the demand for maternal health services. In Cameroon level of underutilisation of maternal health services increased recently.

In fact, births in medical establishments remain low (43 %) in 2006 with differences between the poor (88 %) and non poor (29,5 %) and between the various areas (11,1 % in the North against 69,9 % in the littoral) and between urban (72,4 %) and rural areas (21,6 %). The proportion of childbirth assisted by qualified health personnel fell between 2004 and 2006. At the national level, it passed from 61,8 % in 2004 to 58,9 % in 2006 [21]. The immediate consequence of this low recourse to health services is the rise of maternal mortality which passed from 430 to 669 deaths per 100 000 births for the period 1998–2004 to 782 deaths in 2011. These deaths are closely linked to the frequency of visits to health establishments during pregnancy and at childbirth [21]. The health services received by a mother during pregnancy, at child birth and immediately after are not only very important for her survival and welfare but also for that of the child. In Cameroon, a real competition occurs for the supply of medical services. In fact, the resort to modern medicine is in strong competition with self medication (modern or traditional) and because of the absence of health services, their use is mainly controlled by patients (it reflects individual choices and decisions) and not by health professionals who have only a marginal control on the demand for services and treatment choices of their patients [22]. To be efficient, prenatal care must begin at an early stage of the pregnancy and continue regularly until childbirth. The World Health Organization (WHO) recommends at least four antenatal visits, the first in the third month of pregnancy with regular intervals throughout the pregnancy. Among the women having had a live birth during five years preceding the survey, 62 % had at least the recommended four prenatal visits and this proportion is higher in urban (77 %) than in rural areas (50 %). Concerning child delivery, among the deliveries which occurred during the five years preceding the survey, approximately three fifths occurred in a health establishment (61 %). In 37 % of the cases, the women have delivered at home. Moreover, women of the rural areas (54 %) deliver more frequently at home than those of the urban areas (14 %) [21].

## Methods

The methodology is divided into the theoretical framework, the specification of the econometric model and the justification of the choice of the variables.

### Theoretical framework

The theoretical framework is based on discrete choice models following the examples of [23, 24]. Discrete choice models are derived from the hypothesis that the choice of the individual follows a maximisation behaviour of the random utility [25, 26]. Formally, the utility *U*_*ij*_ that the patient *i* associates to each form of treatment *j* is given by:

1 $$ {U}_{ij}={\beta}_{0j}+{\beta}_{1j}{X}_i+{\beta}_{2j}{Q}_j+{\beta}_{3j}{Y}_i+{\beta}_{4j}{P}_j+{\varepsilon}_{ij} $$

The coefficients *β* are attached to the indices *j* showing that the effects of the explanatory variables can vary according to the treatment route (public, private denominational, private profit seeking and traditional midwives). *X*_*i*_ is the characteristics of the pregnant woman. *Q*_*j*_  *is* the characteristics of the choice of treatment. *ε*_*ij*_ is the random error term which aims at capturing the unobserved determinants of the utility of the pregnant woman *i* who chose the treatment *j. Y*_*i*_ is the income of pregnant woman *i. P*_*ij*_ the total cost of healthcare supported by the pregnant woman *i* who chooses the treatment *j*. The preceding equation can be summarised by:

2 $$ {U}_{ij}={V}_{ij}+{\varepsilon}_{ij} $$

3 $$ \mathrm{With}\kern0.5em {V}_{ij}=\kern0.5em {\beta}_{0j}+{\beta}_{1j}{X}_i+{\beta}_{2j}{Q}_j+{\beta}_{3j}{Y}_i+{\beta}_{4j}{P}_j $$

The probability of selecting treatment route *j* is equal to the probability that the utility given the pregnant woman by the supplier is higher than the utility associated with all the other routes.

That is to say: *P*_*ij*_ = Pr(*U*_*ij*_ > *U*_*ik*_) , ∀ *j* ≠ *k*

4 $$ {P}_{ij}= \Pr \left({\varepsilon}_{ik}-{\varepsilon}_{ij} > {V}_{ij}-{V}_{ik}\right)\kern0.5em ,\ \forall\ j\ne k $$

The calculation of the probability of Eq. (4) depends on the chosen distribution of the error term.

### Econometric specification

**Fig. 1:**
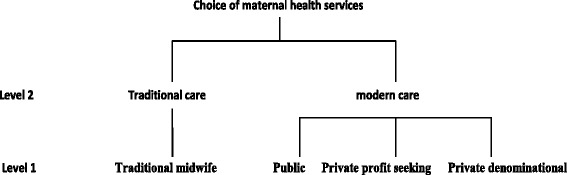
Choice of maternal health services. Source: Authors

The econometric specification depends on the hypotheses made with regard to the distribution of the error term of the probability of Eq. (4). Gertler et al. [23] develop a random utility model in which the demand for medical care is modeled as the decision to seek care and, conditional on that, the decision of which provider to use. The corresponding econometric specification is the nested multinomial logit model, which relaxes the independence of non relevant alternatives (IIA) assumption [27]. The use of a nested logit approach implies that choices can be organized into a meaningful nesting structure. Consider that the J alternatives can be classified in two groups as shown in the Fig. 1.

The expectant woman chooses between traditional and modern services and makes a final choice from the different alternatives in the group chosen. The probability that a pregnant woman choses a service j given that she has chosen to resort to modern health services is given by the equation:

5 $$ Prob\ \left({Y}_i=j/M\right)=\frac{exp\left({\beta}_j^{\hbox{'}}{z}_i\right)}{{\displaystyle {\sum}_k^3} exp\left({\beta}_j^{\hbox{'}}{z}_i\right)} $$

Also, the probability of choosing traditional health services over modern ones becomes:

6 $$ Prob\ \left({Y}_i=0\right)=\frac{exp\left({\beta}_o^{\hbox{'}}{z}_{io}\right)}{exp\left({\beta}_o^{\hbox{'}}{z}_{io}\right)+ exp\left(\lambda .{I}_i\right)},\kern0.5em \mathrm{with}\kern0.5em {I}_i= ln\left({\displaystyle {\sum}_k^3} exp\left({\beta}_j^{\hbox{'}}{z}_i\right)\right) $$

In this formulation, the vector z_io_ corresponds to a set of variables that affect the decision of the woman to resort to traditional or modern health services. The term *I*_*i*_ represents the *inclusive value* that shows the correlation between the choices of the modern health services sub-group. When the parameter λ is different from 1, the nested multinomial logit model drops the IIA hypothesis between the different choices of the decision tree. However, the IIA hypothesis is maintained between choices belonging to the same group. The probability of choosing a modern health service is thus given by:

7 $$ Prob\ \left({Y}_i=j\right) = Prob\ \left({Y}_i=j/M\right).\left(1- Prob\ \left({Y}_i=0\right)\right) $$

### Choice of variables

In the empirical literature, the identification of the determinants of the demand for health services by pregnant women takes into consideration several variables. *The treatment options* are presented in four alternatives: public services, private denominational services, private profit seeking services and traditional midwives. The recourse to public services represents the set of the public health structures. The recourse to private denominational services considers all the denominational private health structures. The recourse to private profit seeking services considers all the private profit seeking services. The traditional midwives takes into consideration the case where pregnant women consult traditional midwives who use grasses and barks of trees. Public services, private missionary institutions and private profit making organisations make up the modern maternal health services while traditional midwives make up the traditional health services. Several independent variables are used to explain the choice of treatment. These variables are explained below. The cost of using the services of health personnel is a factor which can guide the choice to consult a medical personnel or not in case of disease. In fact, when the cost of healthcare increases and the patient does not have the necessary means, she can resort to other alternatives which she finds at her reach or decide not to consult at all [28]. The cost of the healthcare represents the total expenditure on consultation, clinical tests, treatment, drugs and hospitalisation. The household income is one of the significant determinants of the demand of healthcare in a health system insofar as it can increase the probability of using a modern healthcare service. Health is priceless and the lack of a reasonable income cannot deprive a household of quality health services to protect its members. Income is measured in this study by the economic level: very poor, poor, averagely rich and very rich [29].

The perception which the pregnant woman or the members of her household have on the complication of her pregnancy is one of the factors that push pregnant women to demand health services. Women use a healthcare service only when they find that the pregnancy is at risk. If it is not at risk, the woman waits or practices alternative solutions [6, 28]. Medical insurance can also explain the demand for health services because takes care of the costs of treating the insured woman. When one subscribes to insurance, or when we adhere to a mutual health insurance company, we tend to seek treatment in the event of disease near a health professional since we have the assurance that the costs of treatment are taken care of. The variable health insurance covers the insured women and members the mutual health insurance companies. Age has an impact on the choice of medical structures, the older a person is, the greater the probability that he chooses a modern health structure [30, 31]. It is also noticed that the educational level of the mother can be a determinant of the choice of the healthcare service insofar as when one is educated, one is supposed have more information on the importance of visiting a healthcare establishment or consulting a health personnel in case of disease than a person who is less educated [16, 32]. The size of the household is retained because it can influence the decision to seek for care or not. In fact, when a household is large and income is not sufficient, in case of disease, a choice is made between seeking for treatment and providing for other needs of the household. This is not the case when the size of the household is small [33]. Religion affects the attitude of women vis-a-vis pregnancy and modern healthcare. Some religions have a positive impact [34]; while others have a negative impact on the demand for healthcare [35]. There is a relationship between the quality of care and the demand for health services, bad quality reduces the demand for healthcare [36]. The variable quality of care is measured by satisfaction. A sick pregnant woman is satisfied with a provider of healthcare if she is cured by this provider. Transportation cost is not only an additional cost to the cost of healthcare but also an indicator of the proximity of health services. In this study transportation cost reflects the proximity of services and is related to the demand for healthcare [37]. Those who reside in urban environments are closer to healthcare services than those who reside in rural areas [29]. The variables retained from the empirical literature are: the cost of healthcare, the economic level of the household, complications of the pregnancy, health insurance, age, the level of education of the pregnant woman, the size of the household, religion, the quality of the healthcare services, the cost of transportation and place of residence.

### Study data

The data used in this study is from the Demographic Health Survey (DHS) carried out in Cameroon in 2011. This survey is stratified into three main categories: the “Yaoundé/Douala” stratum, the “small towns” stratum and the “rural” stratum. The sampling is done at two levels. At the first level, the zones of sampling (ZS) are drawn with probabilities proportional to the number of households listed in the zones. At the second level, the households are drawn using lists written after an exhaustive enumeration of each ZS. Weighting coefficients are assigned to ensure the auto-weighting of the sample within each stratum. In 2011, the sample consisted of 14214 households, 15852 women aged 15–49 years and 7525 men aged 15–59 years. The survey on maternal health only considers women who requested a follow-up of their pregnancy for their most recent live birth that occurred in the five years preceding the survey. 85 % of women benefit from a follow-up of their pregnancy. Also, the response rate to interviews for these women is 97 %. The DHS carried out in Cameroon in 2011 has information on the person consulted during the pregnancy, the possession of a notebook of antenatal consultations, the duration from the pregnancy to the first consultation, the number of antenatal and postnatal visits, the taking of anti tetanus vaccine and of anti malaria drugs during the pregnancy, the place of childbirth and assistance with childbirth. Table 1 gives the means and standard deviations of the explanatory variables.

**Table 1:** Means and Standard deviations of explanatory variables

Variables	Mean	Standard deviation
AGE		
15-25 years	0,3280	0,4672
26-29 years	0,4413	0,4965
30-49 years	0,2302	0,4209
Level of education		
No education	0,2968	0,4568
Primary education	0,4297	0,4950
Secondary	0,2524	0,4344
Higher	0,0211	0,1436
Occupation		
Paid employment	0,7704	0,4205
Employment not remunerated	0,2296	0,4205
Area of Residence		
Rural	0,5554	0,4969
Urbain	0,4446	0,4969
Religion		
Catholic	0,3439	0,4750
Protestant	0,3380	0,4730
Moslem	0,2344	0,4236
Animist	0,0302	0,1711
Other Christian	0,0228	0,1492
Matrimonial status		
Single	0,0374	0,0189
Married	0,7172	0,4503
Cohabitation	0,1342	0,3409
Widow	0,0552	0,2282
divorced	0,0374	0,1897
Size of household		
1-4	0,2338	0,4232
5-10	0,4 811	0,4933
11 and more	0,3 297	0,3360
State of previous pregnancy		
Complicated	0,03	0,178 4
Noncomplicated	0,97	0,1778
Index of wealth		
Very poor	0,2111	0,4080
Poor	0,2392	0,4266
Average	0,2212	0,4150
Rich	0,1860	0,3891
Very rich	0,14 00	0,350 0
Cost of care		
100-1000	0,2 78 0	0,1323
1005-10000	0,3 83 0	0,0909
10005 and more	0,36 19	0,0436
Cost of transport		
0-100	0, 24 11	0, 4906
150-400	0,41 7 0	0, 2654
500 and more	0, 33 12	0, 5699
Insurance		
Ensured	0,0298	0,4122
Not assured	0,9702	0,7701
Quality of the care		
Bad	0,5841	0,227 8
Good	0,4159	0,49 45

Source: Author using data from [21]

## Results and discussion

Table 2 gives the results of the Nested Multinomial Logit regression. Estimates in Table 2 reveal several lessons. The level of education of the woman has a significant effect on the choice of the type of service. Uneducated women or women having received only a primary education prefer visiting the traditional midwives to modern healthcare services. In other studies [8–13], a similar result is found. The relationship between the access to a remunerated employment for the pregnant women and the use of the modern healthcare services is positive. This result confirms that the decision to choose modern healthcare services to the detriment of the traditional midwives can be explained by the nature of the employment of the pregnant women. This result is confirmed by [11]. However, employment may not necessarily be associated with greater use of maternity care if: (i) women have little control over their earnings; (ii) employment is largely poverty-induced and reflects resource constraints and (iii) employment is seasonal and poorly remunerated [11, 34, 38].

**Table 2:** Results of the estimates of the nested multinomial logit model (marginal effects)

	Traditional midwife		Public		Private denominational		Private profit seeking	
Variables	Dy/dx	P>/Z/	Dy/dx	P>/Z/	Dy/dx	P>/Z/	Dy/dx	P>/Z|
*Choice of modern healthcare services (level 2)*								
Age 15-25 years	ref.	ref.						
26-30 years	-0,052229	0,978	-	-	-	-	-	-
31-49 years	-0,077863**	0,034	-					
Level education				-	-	-	-	-
No education	0,716561***	0,002	-	-	-	-	-	-
Primary education	0,277552*	0,073	-	-	-	-	-	-
Secondary	-0, 8183958**	0,016	-	-	-	-	-	-
Higher	ref.	ref .	-					
Occupation				-	-	-	-	-
Remunerated employment	-0,1025579**	0.033	-	-	-	-	-	-
Employment not remunerated	ref.	ref .	-					
Religion				-	-	-	-	-
Catholic	-0,1585394	0,446	-	-	-	-	-	-
Protestant	-0,446	0,210	-	-	-	-	-	-
Moslem	0,431396*	0,000	-	-	-	-	-	-
Animist	0, 394046 *	0,903	-	-	-	-	-	-
Other Christian religions	ref.	ref.	-	-	-	-	-	-
State of last pregnancy			-	-	-	-	-	-
Complicated	-0,4353877**	0.032	-	-	-	-	-	-
Non complicated	ref .	ref .	-	-	-	-	-	-
Matrimonial statute			-	-	-	-	-	-
Single	0,7689184***	0,001	-	-	-	-	-	-
Married	-0,4279708**	0,010	-	-	-	-	-	-
Cohabitation	-0,1742246	0,302	-	-	-	-	-	-
Widowed/Divorced	Ref.	ref.	-	-	-	-	-	-
Size of the household			-	-	-	-	-	-
1-4	ref.	ref.	-	-	-	-	-	-
5-10	0,4580059**	0.014	-	-	-	-	-	-
11 and more	0,527099***	0.001	-					
Index of wealth				-	-	-	-	-
Very poor	0,430192***	0,000	-	-	-	-	-	-
Poor	0,285039***	0,000	-	-	-	-	-	-
Average	-0,458281***	0.006	-	-	-	-	-	-
Rich	-0,380440**	0,088	-	-	-	-	-	-
Very rich	ref.	ref .	-	-	-	-	-	-
Type of modern healthcare services chosen *(level 1)*								
Cost care (FCFA)								
5000-30000	-	-	ref.	ref.	ref.	ref .	ref .	ref.
30005-100000	-	-	- 0,0726**	0,025	-0,085***	0.003	-0,0222*	0,098
100005 and more	-	-	- 0,26486***	0,000	-0,177***	0,000	-0,009**	0,023
Cost of transport (FCFA)								
0-100	-	-	ref.	ref.	ref.	ref.	ref.	ref.
150-400	-	-	-0,70673**	0,024	-0,50480	0,711	0,0155 0	0,260
500 and more	-	-	-0,781356**	0,032	-0,56054*	0,093	0,5403***	0,000
Insurance								
Ensured	-	-	0,455742***	0,001	0,8821***	0.000	0,7683***	0,000
Not assured	-	-	ref.	ref.	ref.	ref.	ref.	ref.
Quality of the care								
Good	-	-	0,51071**	0,032	0,570***	0,000	0,6287*	0,0820
Bad	-		ref.	ref.	ref.	ref.	ref.	ref.
Place of Residence		-						
Rural	-	-	-0,304343**	0,032	-0,1095*	0,079	-0,0023	0.540
Urban	-	-	ref.	ref.	ref.	ref.	ref.	ref.
*Inclusive value* λ	4,465***	0.000						

Number of individuals = 4485; Wald chi2(124) = 2837.126; Log Prob > chi2 = 0.0000 likelihood = -5366.5916

Source: Author using data from [21]. Note: indication of level of significance: ***p ≺ 0.01, **p ≺ 0.05, *p ≺ 0.1 *ref.: reference class = traditional midwife, for level 1*

The women of Islamic and animist religions are those who prefer the services of the traditional midwives to those of modern medical institutions. A similar result is obtained by [35]. The effect of culture can partially explain this result. In fact, in the North and Far-North regions which are mainly Moslem, the decision to resort to maternal healthcare services does not belong to the pregnant woman but to her husband. In addition, the traditional midwives are closer to the pregnant women than modern services of healthcare which are sometimes too far. In the case of the animist religion, cultural practices also favour the choice of the traditional midwives to modern services of maternal healthcare. Reinforcing the recourse to modern services of maternal healthcare in a context strongly influenced by cultural values inevitably passes through the implication of the traditional midwife to the processes of modern of healthcare [39]. However, the relationship between religion and the demand for modern maternal health services could also be explained by differences in living standards between the different religious groups and not religion. It is possible that women belonging to a particular religious group are poorer and thus have a lower demand for modern health services [40]. In the case of Cameroon, the high rate of poverty in Moslem regions could explain the negative relationship between the Moslem regions and the low demand for modern health services by women from these regions. The women whose last pregnancy was complicated tend to visit modern medical institutions than request the services of traditional midwives. The study by Magadi et al. [6] holds that complications in the first pregnancies have a positive effect on the demand for modern health services. In the same manner, a large size of the household seems to be unfavourable to the use of modern medical institutions by the pregnant women. Furthermore, the married pregnant woman uses modern maternal services more than the single woman. This result is in line with those of [11]. The positive influence of marital status may reflect the stigmatization single expectant mothers go through since childbearing, in most communities, is perceived as an act reserved for those who are married [41]. In addition when a pregnant woman is poor, she prefers the services of the traditional midwife to those of the modern health services. Similar studies [8, 11, 42] also confirm the negative effect of poverty on the demand for modern health services.

Concerning the type of modern health services chosen by pregnant women, many lessons can be drawn. The cost of healthcare discourages the recourse to modern services of healthcare. In fact, when the costs of healthcare increase in modern medical institutions (public, Private denominational, Private profit seeking), the women prefer to use the services of traditional housewives. The costs of transport also constitute a barrier to the use of modern services of healthcare by pregnant women. Other studies [8, 43] agree that the cost of maternal health services and transportation costs are financial obstacles faced by pregnant women who decide to use modern maternal health services. Also, maternal health units that offer services covered by a health insurance attract more pregnant women. The quality of services is also a factor that affects the choice of a healthcare provider. Our results show that the positive effect of the quality of healthcare on the choice of healthcare provider is higher for private profit seeking and denominational hospitals. A similar study [44] confirms that pregnant women use the services of a given provider when the perceived quality of treatment meets expected standards in past visits. We also find a negative relationship between rural maternal health service providers and the use of these services by pregnant women. This negative relationship can be explained by physical obstacles, particularly transport faced by rural women who are generally poorer [8, 11].

However, this study is limited to the factors explaining the choice of maternal healthcare services by pregnant women. Specifically, the study does not investigate the determinants of the volume of maternal healthcare services consumed once the provider has been chosen. Meanwhile, after choosing the maternal healthcare service provider by the pregnant women, it is important to analyze the factors that determine the quantity of the maternal healthcare services they consume. As such, uture studies would aim at identifying the factors that determine the quantity of healthcare services consumed once the provider has been chosen. This is important as access to maternal healthcare services is not limited only to the choice of the healthcare service provider.

## Conclusion

This study seeks to identify the determinants of the treatment choices of pregnant women in Cameroon. Among the significant results of the empirical analysis, we can retain that the absence of education or primary education are factors which reduce the demand for modern services of maternal health on the one hand and on the other hand favour the demand of the services of traditional midwives on the other hand. Moreover, the absence of a paid employment for the woman, the large size of the household, Islamic or animist religions, the poor quality of healthcare services in modern healthcare centres, poverty, high costs of healthcare and transport are constraints which push pregnant women to prefer the services of traditional midwives to those of the modern services of maternal health. From the results of this study, three important lessons are retained: firstly, the education of women increases their capacity to use modern maternal healthcare services. The low observed school attendance rate girls therefore partly explains the high maternal mortality rate observed in certain regions like the North and Extreme-North regions of the country. Secondly, salaried employment enforces the economic power of women, increasing their capacity to use modern than traditional maternal healthcare services. Therefore, maternal mortality can also be reduced through the economic empowerment of women. Finally, the existence of a health insurance scheme increases the use of modern maternal healthcare services. In fact, healthcare services directly financed by pregnant women or their families can be a factor of exclusion especially for poor women. After choosing the maternal healthcare service provider by the pregnant women, it is important to analyze the factors that determine the quantity of the maternal healthcare services they consume. As such, future studies would aim at identifying the factors that determine the quantity of healthcare services consumed once the provider has been chosen.
